# Editorial

**Published:** 1872-08-15

**Authors:** 


MEDICAL EXAMINER.

*A Semi-Monthly Journal of Medical Sciences.*

EDITED BY

N. S. DAVIS, M. D., AND F. H. DAVIS, M. D.

Chicago, August 15th, 1872.

### EDITORIAL.

How Not To, and Why.—During the early part of the present year, a work was published on the hygienic management and proper physical training of children, written by Dr. Dan Newcomb, and designated "*When and How.*" Although we did not deem the style of the author wholly unexceptionable, yet the facts and principles inculcated in that work were correct, and were calculated to do good. We consequently noticed and freely commended the book to the patronage of the public. Since the publication of that work, the same author has caused to be published another and much smaller work entitled "How Not To, and Why; or, Arguments based on Physiological, Moral, and Social Relations in favor of Preventing Conception, and the giving of the *Ways and Means* in Plain Language." We find that some persons have mistaken our favorable notice of the former work in the Examiner as applying to the latter. To do justice, therefore, both to ourselves and to the public, it becomes necessary to say what we think of the author's last work. It is founded on three leading ideas:

First, That excessive child-bearing, at the present day, constitutes a great physical and social evil; by injuring physically the mother, impairing the physical strength of the offspring, and bringing more children into the world than the parents can properly provide for and educate.

Second, That men are beastly and unconquerable in their sexual passions, and will not practice continence, and therefore the woman should have the right to control the number of her children, and to that end should know the means for preventing conception.

Third, That to a great extent, at least, parents can control both the sex and the mental and physical character of their offspring, by observing certain rules and modes of life.

In support of the first idea, the author gives no facts, no statistics, but simply represents the condition of pregnancy as one of constant and unmitigable suffering, and nursing as one of injurious exhaustion. He deals in such general expressions as the following:

"They are filled with suffering all through the days of the intra-uterine life of the child, and end that stage in pangs of suffering that cannot be expressed in language."

Again, he says:

"Yet, though the mother bears every pain, performs every labor—in fact, does everything necessary to rear a child—we hear it said that she should not have a voice in the number she shall raise; but rather submit herself to the embraces of a brutal, beastly, passionate husband, who has only a pleasurable part to perform, as often as he may desire, without any power to prevent conception."

These arguments (so-called) in favor of woman's right to determine the number of her offspring, are simply appeals directly calculated to raise prejudice between the sexes, and to alienate the wife from her husband. Thus, after detailing all the horrors, mental and physical, accompanying the pregnant state, he exclaims:

"Who bears all this? The mother. And what part does the father have in the labors of raising children? Simply nothing. He is only necessary in the raising of children to assist in the creation, or the conception."

And again:

"While woman does the work and suffers the pains, she should have a voice in the frequency of those labors and pains. And if she must submit to the embraces of her husband, she should know how to prevent a conception following the favor."

After describing the sexual organs in both sexes and the part each sex performs in procreation, in the first chapter; giving a nauseating amount of the stuff we have quoted, in the second; and in the third mixed in a little moral seasoning in the way of condemning abortions and abortionists, he proceeds to give the *means* by which the married woman is to protect herself from the dire calamities of over-child bearing. On the title page he promises to give the "Ways and Means in Plain Language." And from the general tenor of the work, the readers' expectations will be raised to a high point in regard to these ways and means. And after all, what are they? Simply two. The first is the pretended physiological fact that there is a period of from seven to ten days, beginning twelve days after a monthly period and ending three days before the next one, during which the most free sexual intercourse may take place without danger of conception. This period he thinks is sufficient for all proper gratification of the sexual passions of the sexes. If it is true that such a physiological law exists, which we very much doubt, and both men and women were so considerate and strictly continent as to keep the times and seasons fully in memory, it might be available in regulating the number of children. But if the law was ever so reliable, and man as passionate and incontinent as our author represents him to be, of what use would it be to the poor wife. How is she to limit her "brutal, beastly, passionate" husband to those few days in each month? Admitting the difficulty of accomplishing this, the author gives one other remedy, viz.: cold water injections used immediately after sexual intercourse. This, though admitted to be very unpleasant to the woman, is claimed to be, both effectual and free from danger, *if used properly.* But what constitutes the *proper* use? Just how many minutes may safely be allowed to pass after the conjugal act is completed before the cold water must fill the vagina? How cold must the water be, and what quantity must be used to make the remedy certain?

Direct and exact answers to these questions, in "plain language," is just what the reader would expect the author to give. But all the answer he sees fit to give is the following: "We know of hundreds who have used it for years, and never found it to injure them in any way. *Be sure you know how to use it, and then you are safe.* For more *specific advice*, we refer you to your *physician.*"

Imagine the expression that would come over the countenance of one of those young women, either just married or just about to be, who would rather spend the first half-dozen years of her married life in boarding-house flirtations, and at theatrical exhibitions, than in making herself mistress of a home and family circle of her own, when, after anxiously reading through all the rest of this book, she comes to the last paragraph just quoted. To come to the gist of the whole matter, the very thing anxiously sought, the remedy safe and certain, if used at the *right time* and in the *right way*, and then, for the details of the *time* and *way*, to be referred to the family physician, is much like being placed in the position of the fabled Tantalus. Certainly, if the author "knows of hundreds who have used it for years, and never found it (the cold water injection) to injure them in any way," he ought to know just *when* and *how* it should be used; and his failure to give these directions is a cowardly skulking from the responsibility of doing just what he fairly promises, both in the title-page and general tenor of his book. It is very easy to say a remedy is safe and certain, if *properly* used, but leave the directions as to what would be the *proper* use to somebody else. Then, no matter how many may make themselves sick, or even produce death, the author can always screen himself behind the assertion that the remedy was *not* properly used. We have practised medicine several years longer than the author of this little work, and have been brought in contact with a large number of married women, but so far from having found *hundreds* who had used the cold water injection successfully for years in preventing conception, we have not in the whole time found a single score who acknowledged that they had used it half a dozen times each. And yet, of the limited number we have met, several made themselves severely sick. One young married woman, during the last twelve months, in the frequent use of decidedly cold water immediately after the sexual intercourse, brought upon herself a metritic and vaginal inflammation, so acute and severe as to endanger her life, and yet she did not prevent conception, but, on recovering from her severe illness, found herself pregnant, and at the proper time since has been safely delivered of a healthy child. And I may add that she is now both healthier and happier than she would be if she was still fighting off conception with cold water injections. The truth is, this little work of Dr. Newcomb's is one of a class of purely sensational works. He exaggerates immensely the frequency of the practice of criminal abortion; exaggerates still more the evils of pregnancy and child-bearing; fosters that sickening, mawkish sentimentality about child-bearing and the relations of man and wife, which has already done much to corrupt public sentiment, by lessening the idea of sacredness and value in the individual home and family, while its pretended remedies are delusive and dangerous. We have no doubt but the author is honest in the sentiments he utters, and intended his work should benefit society. But believing him entirely mistaken, and having our name connected with his previous work on the proper training of children, we felt under obligations to express a decided disapproval of the volume under consideration.

